# Lack of Association of Vascular Risk Factors with HIV-Associated Neurocognitive Disorders in cART-Treated Adults Aged ≥ 50 Years in Tanzania

**DOI:** 10.3390/v16060819

**Published:** 2024-05-22

**Authors:** Katherine A. Flack, Emma S. Rainey, Sarah J. Urasa, Sengua Koipapi, Rajesh N. Kalaria, William P. Howlett, Elizabeta B. Mukaetova-Ladinska, Marieke C. J. Dekker, William K. Gray, Richard W. Walker, Catherine L. Dotchin, Himidi Mtwaile, Thomas C. D. Lewis, Lydia G. Stone, Richard J. Q. McNally, Philip C. Makupa, Stella-Maria Paddick

**Affiliations:** 1 Faculty of Medical Sciences, Newcastle University, Newcastle upon Tyne NE2 4HH, UK; 2 Kilimanjaro Christian Medical University College, Moshi, Kilimanjaro PO Box 2240, Tanzania; 3 Department of Neuroscience, Behaviour and Psychology, University of Leicester, Leicester LE1 7HA, UK; 4 The Evington Centre, Leicester General Hospital, Leicester LE5 4QF, UK; 5 Department of Medicine, Northumbria Healthcare NHS Foundation Trust, North Tyneside General Hospital, North Shields NE29 8NH, UK; 6 Department of Radiology, NSK Hospital, Arusha P.O. Box 3114, Tanzania; 7 Department of Old Age Psychiatry, Cumbria Northumberland Tyne and Wear NHS Foundation Trust, Newcastle upon Tyne NE4 6BE, UK; 8 HIV Care and Treatment Centre (CTC), Mawenzi Regional Referral Hospital, Moshi, Kilimanjaro P.O Box 3054, Tanzania; 9 Department of Old Age Psychiatry, Gateshead Health NHS Foundation Trust, Gateshead NE8 4YL, UK

**Keywords:** HIV, vascular risk factors, HIV-Associated Neurocognitive Disorders (HAND), older adults, sub-Saharan Africa, cognitive impairment

## Abstract

HIV-associated neurocognitive disorders (HAND) are highly prevalent in those ageing with HIV. High-income country data suggest that vascular risk factors (VRFs) may be stronger predictors of HAND than HIV-disease severity, but data from sub-Saharan Africa are lacking. We evaluated relationships of VRFs, vascular end-organ damage and HAND in individuals aged ≥ 50 in Tanzania. c-ART-treated individuals were assessed for HAND using consensus criteria. The prevalence of VRFs and end organ damage markers were measured. The independent associations of VRFs, end organ damage and HAND were examined using multivariable logistic regression. Data were available for 153 individuals (median age 56, 67.3% female). HAND was highly prevalent (66.7%, 25.5% symptomatic) despite well-managed HIV (70.5% virally suppressed). Vascular risk factors included hypertension (34%), obesity (10.5%), hypercholesterolemia (33.3%), diabetes (5.3%) and current smoking (4.6%). End organ damage prevalence ranged from 1.3% (prior myocardial infarction) to 12.5% (left ventricular hypertrophy). Measured VRFs and end organ damage were not independently associated with HAND. The only significant association was lower diastolic BP (*p* 0.030, OR 0.969 (0.943–0.997). Our results suggest that vascular risk factors are not major drivers of HAND in this setting. Further studies should explore alternative aetiologies such as chronic inflammation.

## 1. Introduction

There are currently an estimated 38 million people living with HIV (PLWH) worldwide, over two-thirds of whom reside in sub-Saharan Africa (SSA) [1]. Combined antiretroviral therapy (cART) has near-normalised life expectancy with HIV, but has resulted in additional challenges from age-related non-communicable diseases (NCDs) [2,3]. NCDs, including cardiovascular disease (CVD) and diabetes mellitus (DM) appear to occur earlier and more frequently among PLWH compared to age-matched controls [4,5]. Furthermore, the chronic complications of HIV, such as HIV-associated neurocognitive disorders (HAND) are increasingly recognised [6,7].

HAND include a spectrum of disorders including asymptomatic neurocognitive impairment (ANI), mild neurocognitive disorder (MND) and HIV-associated dementia (HAD) [6]. Post introduction of cART, HAND prevalence appears to have increased, but milder clinical phenotypes (ANI, MND) now predominate [8,9,10]. Older age appears to increase the risk of cognitive impairment in PLWH, suggesting that HAND prevalence may increase as PLWH age [11,12]. It is predicted, therefore, that HAND will become a major cause of cognitive impairment in SSA [13]. The pathophysiology of HAND is poorly understood. Current aetiological hypotheses include direct neurotoxicity from HIV proteins, the effects of chronic central nervous system (CNS) inflammation and neurotoxic effects of cART [14,15].

Recent data from both high-income countries (HICs) and SSA suggest that HAND are not associated with HIV-disease severity factors in cART-treated PLWH [16,17,18]. There is a growing consensus that vascular damage may represent one of the main drivers of HAND in the cART era [19]. Several large HIC studies report vascular risk factors (VRFs) to be stronger predictors of HAND than HIV-disease severity [16,20,21]. In SSA, VRFs, and cardiovascular disease (CVD) are increasingly prevalent in the general population, and a major public health concern [22,23]. Moreover, HIV infection is consistently associated with increased CVD risk including stroke in HIC globally, and in studies from SSA [24,25,26]. Similar associations are reported in other chronic inflammatory disorders (e.g., rheumatoid arthritis) [27].

Given the potentially modifiable nature of VRFs, data examining the relationship between VRFs, CVD and HAND in SSA are needed. Our aim was, therefore, to evaluate the potential relationships between VRFs, vascular end-organ damage (EOD) and HAND in older cART-treated PLWH under regular follow up in Tanzania.

## 2. Materials and Methods

Participants were recruited from Mawenzi Regional Referral Hospital (MRRH) HIV Care and Treatment Centre (CTC), in Kilimanjaro, Tanzania, where 1361 PLWH aged ≥ 50 years were registered on 1 March 2019. As per 2017 national guidelines [28], cART is offered from diagnosis (pre-2017, CD4 count < 350 cells/μL or WHO HIV stage 4) with ongoing subsequent 3-month review. The standardised individual HIV-disease outcome measures are recorded from diagnosis onwards.

### 2.1. Ethical Consideration

The Kilimanjaro Christian Medical University College Research Ethics Review Committee and Tanzanian National Institute for Medical Research approved this study. Participants were given written and verbal information by trained staff, before indicating informed consent. Where participants lacked capacity, due to cognitive impairment, assent was sought from a close relative. Participants and their peers were assured that study withdrawal or refusal would not influence clinical care. Further informed consent was sought to contact an informant for collateral history of cognitive and functional impairment without disclosing HIV status. The results of vascular evaluation were shared with patients verbally and in writing and placed in clinical records, and onward referral pathways were agreed with local clinics.

### 2.2. Sampling and Baseline Data Collection

Data were collected over 11 weeks, from March–May 2019. Individuals aged ≥ 50 years attending routine follow-up were systematically sampled from each routine weekday clinic (excluding public holidays) in order of arrival and registration at the clinic. Sampling frequency was 1-in-3 (every third eligible individual approached in sequence). Where lower attendance was predicted by senior nursing staff (e.g., due to weather conditions), sampling frequency was 1-in-2. Exclusions were individuals attending unscheduled emergency appointments, those who were acutely unwell, or where participation might delay necessary treatment.

### 2.3. Demographic and HIV-Disease Data

Self-reported demographic data included: age, sex, occupation, current employment, education level, marital status and living arrangements. Birth year was crosschecked with hand-held clinic registration cards.

HIV-disease parameters obtained from standardised clinic data sheets included: date of diagnosis, nadir CD4 count, WHO HIV stage, previous and current cART regimen and history of TB or empirical treatment of CNS infection. An additional current CD4 count and HIV viral load (VL) were obtained at assessment. Viral suppression was defined as <20 copies/mL. The recorded WHO HIV stage was reclassified following clinical assessment and case note review [29]. Medication concordance was self-reported.

### 2.4. Assessment of Vascular Disease and Risk Factors

#### 2.4.1. Vascular Risk Factors (VRFs)

Height and weight were measured using a stadiometer and the clinic weighing scale. Body Mass Index (BMI) was calculated as weight/height^2^, and individuals were classified using WHO criteria as underweight (<18.5 kg/m^2^), normal weight (18.5–24.9 kg/m^2^), overweight (25–29.9 kg/m^2^) or obese (≥30 kg/m^2^) [30]. Waist-to-hip ratio (WHR) was measured and abdominal obesity reported using WHO-recommended WHR thresholds (≥0.90 male, ≥0.85 female) previously used in SSA [31,32]. Systolic and diastolic resting BP was recorded 3 times, 5 min apart, and the mean of the second and third readings recorded (WHO STEPS protocol) [33].

Hypertension was defined using WHO criteria (systolic BP ≥ 140 mmHg and/or diastolic BP ≥ 90 mmHg) [34] and/or current antihypertensive prescription. Diabetes mellitus (DM) was self-reported. Hypercholesterolemia was defined as ≥5.2 mmol/L total serum cholesterol (TSC), a cut-off frequently used in SSA [35]. Self-reported tobacco smoking was categorised (never, past, or current smoking). Alcohol intake was self-reported (never, previous, occasional, and regular (weekly consumption)).

#### 2.4.2. Vascular End Organ Damage (EOD)

The markers of vascular end organ damage were measured as follows. The resting 12-lead electrocardiogram (ECG) was recorded using a MAC 1200 ST. The visual abnormality assessment (using KF) included atrial fibrillation (AF) and left ventricular hypertrophy (LVH) using Sokolow–Lyon criteria [36]. Prior MI was defined using WHO criteria (any Q-wave ≥ 0.02 s, QS complex in V2–V3, or Q-wave ≥0.03 s and ≥0.1 mV deep or QS complex in I, II, aVL, aVF or V4–6 in two contiguous leads) [37]. The ankle-brachial pressure index (ABPI) was calculated by dividing the highest ankle BP by the highest brachial BP measured by a Diaped flux-2000 vascular Doppler. An ABPI ≤ 0.90 was defined as peripheral arterial disease (PAD) [38], while ABPI ≥ 1.30 was considered suggestive of arterial stiffening [39]. The pulse pressure was calculated as a surrogate measure of arterial stiffness [40]. Siemens Multistix 10 SG were used to identify proteinuria, classified as ≥1 plus protein on urine dip. Proteinuria was used as a surrogate marker of renal EOD as in other low-resource settings [39]. Calculated eGFR ≤ 60 μmol/L was used to define chronic kidney disease (CKD), as per Kidney Disease Improving Global Outcome staging [41]. A previous stroke was defined via self-reporting, and/or neurological examination findings of an upper motor neuron lesion or an infarct visible on MRI brain imaging (where available).

Retinal photographs were obtained using a VOLK InView camera, at an additional appointment. Pupils were dilated with topical tropicamide 1%. ImageJ software was used to report arterio-venous ratios (AVRs) as a marker of hypertensive retinopathy [42]. The width of paired superior-temporal and inferior-temporal vessels were measured linearly from the fovea to the disc centre, for standardisation. The mean was calculated for each eye, and the mean of these was used to produce one overall ratio. Measurements were repeated to reduce error, averaging initial and repeated measurements, and to assess intra-observer reliability. A medical retina research fellow (LS) blindly reported 20% of images for inter-observer reliability.

### 2.5. MRI Assessment

A demographically representative subset, (the first *n* = 100 systematically recruited), were evaluated with a 1.5T MRI brain. A local radiologist with neuroradiology experience (HM) clinically reported the images. Large-vessel infarcts or haemorrhages were reported and recorded. White matter disease (WMD) was clinically rated mild, moderate, or severe. The pre-agreed protocol for the referral of individuals with unexpected findings was for neurosurgical referral, and participants were counselled regarding this possibility during the consent process.

### 2.6. HAND Diagnosis and Classification

HAND were diagnosed by consensus panel (EML, S-MP, TL) based on the American Academy of Neurology (Frascati) criteria using a standardised protocol and case summary previously described in detail [6,43]. Cognitive impairment was assessed using a locally normed low-literacy neuropsychological battery of cortical and subcortical tests [43,44], (see Figure S1, Supplementary Data) administered by trained nurses and research assistants following a one week harmonisation and training update. Other potential causes or confounders of cognitive impairment were identified through standardised mental state examination, the Geriatric Depression Scale-15 (GDS-15) [45] and the depression and suicidality modules of the Mini International Neuropsychiatric Interview (MINI) [46]. The Confusion Assessment Method (CAM) was used to screen for delirium [47]. A targeted neurological examination assessed for abnormalities including previous stroke and Parkinsonism. Visual acuity was measured with a Landolt C low-literacy logmar distance chart, as a confounder of neuropsychological test performance.

The functional impairment (for identification of symptomatic HAND) was assessed through the Barthel Index, the Karnofsky performance status [48], the self-reported questionnaire and the structured informant history of cognitive and functional impairment, including locally validated instrumental activities of daily living (IADL) screen [49].

### 2.7. Statistical Methods

Data were analysed using IBM SPSS version 26.0. Data were visually examined for normality using histograms and standard descriptive statistics (mean, standard deviation (SD), median interquartile range (IQR), frequency and percentage) were used for normally distributed and non-normally distributed data, respectively. We calculated 95% confidence intervals for prevalence. With regard to the primary and secondary outcomes (HAND versus no HAND, and symptomatic HAND versus ‘no symptomatic HAND), between-group statistical comparisons were examined using Pearson chi2 for categorical variables, with Fisher’s correction for lowest expected cell values <5. Independent t-tests were used to compare normally distributed variables and the Mann–Whitney U test was used for non-normally distributed and ordinal data. These were examined for demographic, HIV-disease factors, VRFs and EOD markers. The relationships between variables significant on bivariate analysis, potential sociodemographic confounders (age and sex), and both HAND (primary outcome) and symptomatic HAND (secondary outcome) were examined using binary logistic regression. The intraclass correlation coefficient (ICC) estimates were calculated for the initial and repeat measurements based on an absolute-agreement, 2-way mixed-effects model. Two-tailed tests and a significance of <0.05 were used throughout.

## 3. Results

**Figure 1:**
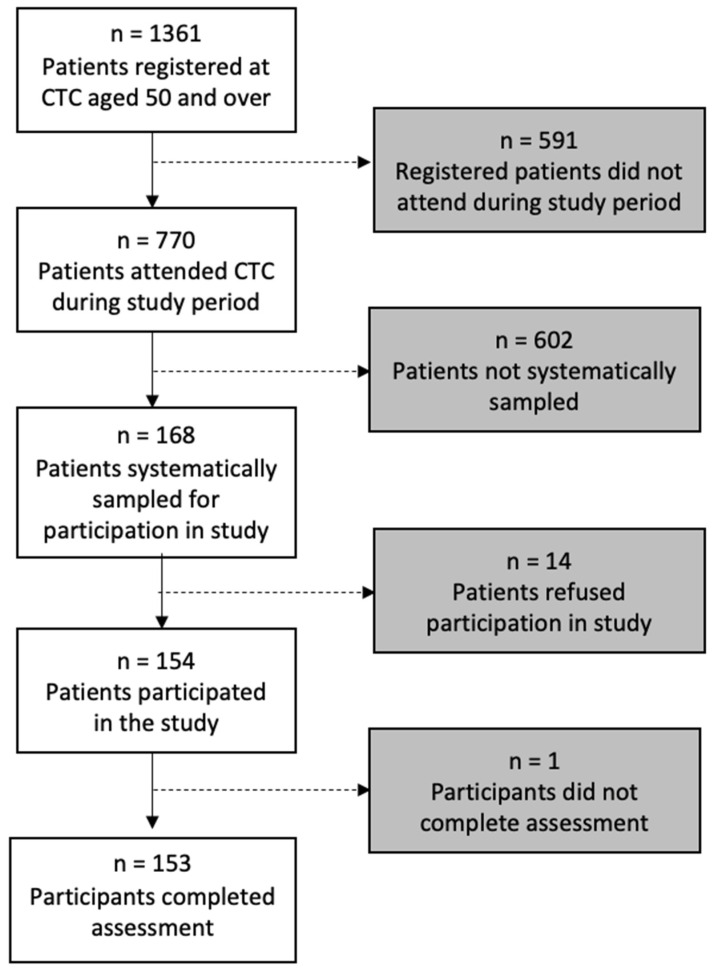
Study flowchart.

The complete data were available for 153 individuals (study flowchart, Figure 1). Sociodemographic and HIV-disease characteristics are listed in Table 1. HIV-disease appeared as a well-controlled (70.5% (*n* = 105) undetectable viral load, with a mean of CD4 499.8, SD 269.6 cells/mm^3^), and a median years post-diagnosis of 11 (IQR 6.0–13.0).

### 3.1. Vascular Disease Burden

The prevalence of VRFs and EOD markers are summarised in Table 2 and VRFs by age group in Table 3. Hypertension was significantly more prevalent among older participants, but other VRFs were not. Notably, of 90 individuals with MRI brain, three strokes were visible on MRI but only one self-reported. Conversely, two participants without visible stroke on MRI reported previous strokes.

For those with retinal imaging (n-130), the mean AVR was 0.75 ± 0.08. The ICC for initial/repeat measurements was excellent at 0.959 (95% CI 0.942–0.972) and the ICC for average measures (subsample *n* = 25, blind independent ratings) was good (ICC 0.880, 95% CI 0.736–0.946). The average AVR was moderately correlated (Pearson’s test) with the systolic BP, r = −0.310, *p* = 0.001, diastolic BP, r = −0.305, *p* = 0.001, and hypertension, r = −0.399, *p* = < 0.001, supporting an increased arterial narrowing in hypertension.

Subjective WMD severity ratings were reported for 90 patients without a substantial MRI movement artefact and complete clinical data (one was excluded due to large incidental meningioma and was referred to neurosurgery). WMD was clinically reported in 57% (20% mild, 36% moderate, 1% severe).

### 3.2. HAND Prevalence

Overall HAND prevalence was high at 66.7% (95% CI 59.2–74.1, *n* = 102), of whom 41.2% (95% CI 33.4–49.0, *n* = 63) met ANI criteria and 31.4% had symptomatic HAND, including 23.53% MND (95% CI 16.8–30.2, *n* = 36) and 1.96% HAD (*n* = 3).

### 3.3. Associations of HAND with HIV-Disease Factors

There were no significant associations when comparing HAND versus no HAND (primary outcome) and symptomatic HAND versus ‘no symptomatic HAND’ (secondary outcome) on any of the measured HIV disease factors including current and nadir CD4, time since diagnosis, HIV disease stage, or HIV viral load (see Supplementary Data, Tables S1 and S2).

### 3.4. Associations of HAND with Vascular Risk Factors and Vascular EOD

Lower mean diastolic BP was significantly associated with HAND (*p* 0.026, Table 4) and this association remained significant after adjustment for age and sex using a binary logistic regression (OR 0.969 95% CI 0.943–0.997, *p* 0.030) (for model, see Supplementary Data). Hypertension, obesity, abdominal obesity, median BMI, and hypercholesterolemia were numerically higher in those without HAND but non-significant on bivariate analysis (Table 4).

Those with and without symptomatic HAND were compared using identical bivariate analysis. A lower mean diastolic BP (T = 0.950, *p* = 0.046) and a lower mean serum cholesterol (T = 2.342, *p* = 0.021) were significantly associated with symptomatic HAND on bivariate analysis (Supplementary Data Table S2) but were non-significant after adjustment for age and sex (for model, see Supplementary Data Tables S3 and S4).

## 4. Discussion

This is the first study investigating vascular associations for HAND in older PLWH in SSA. Hypertension was less prevalent than rates reported in Tanzanian rural elders (69%, age ≥ 70) [50] and urban older adults (48%, age ≥ 55) [51], but broadly similar to adult ED attendees (34.3%) [52] and SSA-wide estimates for adults of all ages (30%) [53]. This is surprising given that in both high- and low-income settings older PLWH and those receiving c-ART appear to have higher hypertension prevalence [54,55]. However, notably, global meta-analytic data suggest a lower hypertension prevalence in adult PLWH in SSA, though available data are heterogeneous and difficult to compare [55]. Our reported hypertension prevalence is similar to that reported in a recent local study with a younger population (median age 45, 95% virally suppressed, 36.5% hypertensive) [56]. A partial explanation may be that advanced HIV at diagnosis appears to reduce the risk of hypertension [54,57] and HIV stage and weight may confound HIV status. The median CD4 was <200 at diagnosis, suggesting late presentation to services, potentially due to HIV-associated stigma [58]. A further potential explanation could be that PLWH access regular medical care that is not available to the general population. The proportion self-reporting current antihypertensive treatment was 10/54 (18.5%), substantially higher than reported in Tanzanian rural elders (6.1%) [50]. This finding may not be generalizable, since recent local data report that hypertension and diabetes were not routinely screened in HIV care, and that awareness and/or previous diagnosis of hypertension and diabetes were low in younger PLWH [56].

We found a prevalence of vascular EOD markers including prior MI, PAD, CKD and LVH to be low in comparison to previous SSA data [59,60,61,62,63], despite clear associations of these vascular pathologies and HIV in both HIC and SSA settings [64,65,66]. This may correspond with lower-than-expected hypertension prevalence.

However, MI prevalence may have been underestimated due to both the low sensitivity of reporting prior MI from ECG [67] and retrospective reporting, given the high mortality of acute MI in Tanzania [68]. Similarly, none of the 152 individuals completing ECG had AF, in contrast to high AF rates in HIV reported elsewhere [69,70]. An explanation for both findings might be the “healthy survivor” effect, since a 53.3% 1-year AF mortality rate has been previously reported in Tanzania [71]. Moreover, black Africans may more commonly present with paroxysmal rather than persistent AF. The use of one 12-lead ECG reading may have underestimated AF prevalence. [72]. The low prevalence of MI on ECG may correspond with the low prevalence of PAD. PAD is strongly associated with atherosclerosis and is predictive of cardiovascular mortality, and symptomatic PAD is considered a marker for subclinical CVD [73]. This low prevalence is interesting since high rates of stroke incidence are reported in Tanzania [74], though previous prevalence figures are low [75] likely due to high case fatality rates [76].

Over half those completing an MRI brain had WMH (>1/3 rated ‘moderate’). Comparable data are limited due to well-recognised methodological differences and a lack of African data, but increasing WMD severity is a well-recognised risk factor for stroke and cognitive impairment in both HIC and LMIC (non-HIV) settings. Aetiology includes endothelial dysfunction, blood–brain barrier disruption or a combination of both [77]. Comparable HIV-specific data are few, but a US study in younger PLWH with a high rate of uncontrolled HIV (40% Black, 41% pre-cART, median age 42) reported 24% had clinically significant WMD (68% any lesions) [78]. Conversely, 19.6% of individuals presenting with cerebrovascular disease in a Kilimanjaro tertiary care centre were HIV positive (median age 43) [79]. Non-HIV community data, e.g., an Austrian stroke prevention study (*n* 344), report a prevalence of 4.9% moderate and 2.6% severe WMD (age 55–64) [80], and the Mayo clinic (*n* 1462) reported 10.7% abnormal WMD in individuals without dementia aged 50–89 [81].

Stroke cases were potentially higher than those reported in SSA (non-HIV) studies [22], though few methodologically rigorous community studies exist [82]. A much older Tanzanian study (2000) reported a stroke related disability of 0.56% (556 per 100,000, aged ≥ 55) [75]. HIV is a well-recognised independent stroke risk factor [83,84,85] in both HICs and LMICs, but the strength of this relationship in individuals with viral suppression is uncertain. Furthermore, cART protease inhibitors are associated with stroke [86]. We can draw only limited conclusions due to the small sample, low case numbers, and the methodological limitations of self-reporting, illustrated by the poor agreement between self-reported and MRI-confirmed stroke. The most common cause of stroke in HIV is atherosclerosis, including large-artery atherosclerosis (LAA) and small vessel disease (SVD). Existing limited data are inconsistent. HIC studies report LAA to be more likely in virally suppressed patients with traditional VRFs, and SVD more likely in those with ongoing inflammation and an uncontrolled disease. Other studies report LAA to be more likely in those with a lower nadir CD4, and a longer duration of infection [83,87]. Our low prevalence of VRFs and substantial WMD burden suggest a need for a further exploration of SVD and inflammation in HAND risk. Furthermore, a low prevalence of carotid atheroma on Doppler measurement is reported in incident stroke cases in Tanzania [88].

Metabolic risk factors appeared more prevalent than expected. Dyslipidaemia prevalence was higher than in a meta-analysis of African data [35] and with over twice the rates reported in Malawi (95% on cART) [57]. A potential explanation might be the high proportion of PLWH (>one fifth) prescribed second-line cART regimens, often including protease inhibitors with known associations with metabolic abnormalities [89,90]. However, TSC was lower in those prescribed second-line regimens. A substantial proportion were overweight and obese (26.8% and 10.5%, respectively) which is a higher proportion than reported in HICs and some SSA HIV studies [57,91,92], though obesity is a well-recognised growing burden in PLWH in SSA (particularly females) [93].

The mean BMI was similar to current Tanzanian estimates, but obesity was substantially lower than reported in younger Tanzanian PLWH (19.8%) in Kilimanjaro [56]. However, over half met abdominal obesity criteria, arguably a better predictor of CVD than BMI [94]. This might be due to a potential preference for being overweight, given the widely known stigmatising association of being underweight and living with HIV in SSA [95], but this may not be a universal factor since reported obesity prevalence was 26.9% amongst PLWH in Malawi [57].

The HAND prevalence was high (66.7%) in comparison to large HIC cohorts [8,96]. SSA HAND prevalence studies are difficult to compare as the reported prevalence varies widely due to population and methodological differences and the studies of older PLWH on treatment are limited [78,97].

Our overall lack of independent association between measured VRFs and EOD markers (including WMD and stroke) potentially contradict the hypothesis that VCI is the underlying pathological mechanism for HAND [19] in this setting. Though comparable SSA data are lacking, an independent association is reported in some HIC studies, though not others. [21] Chronic HIV infection independently promotes accelerated atherogenesis and endothelial dysfunction. Late presentation, suggested in this cohort by low median nadir CD4, may result in a prolonged high HIV VL exposure and chronic inflammation. Chronic inflammation may contribute to cerebrovascular atherosclerosis and white matter injury, despite the low prevalence of atherogenic risk factors [98]. The lack of association between CVD and HAND in this cohort makes this less likely. Moreover, retinal vasculature is closely associated with cerebral microvasculature. The lack of correlation between retinal arterial narrowing and HAND suggests an alternative explanation than VCI.

An association between diabetes and HAND is reported in HIC studies [99,100], and hyperglycaemia may contribute to non-HAND cognitive impairment [99]. DM was more prevalent in symptomatic HAND (10.6 versus 2.9%), a statistically insignificant difference. Although diabetes was self-reported, rates were similar to the locally reported HIV data supported by laboratory measures [56]. The potential impact of diabetes on HAND warrants further study given the rapidly increasing prevalence in SSA [101].

Contrary to our hypothesis, HAND was independently associated with lower diastolic BP, and in symptomatic HAND, only age and lower TSC approached significance (*p* 0.051 and 0.052, respectively) when adjusted for confounders. These are interesting findings, as we previously reported an association with lower TSC and BP in neurodegenerative dementia amongst rural elders in Kilimanjaro [102].

In our cohort, the measured VRFS and end organ damage markers were not associated with HAND. These findings are perhaps surprising in the context of the evidence of the relationship between both cardiovascular and cerebrovascular diseases and HIV seropositivity [103], and the relationship of vascular disease and HAND [21]. These systematic reviews and meta-analysis largely evaluate studies in HICs. Chronic inflammation can result in endothelial dysfunction (ED), and it may be that traditional lifestyle VRFs do not entirely reflect chronic inflammatory endothelial effects in our cohort. ED is hypothesised to lead to atherosclerosis due to impaired vasoregulation during chronic inflammation [98]. A USA study from the TMARC group [104] found that although PLWH had elevated markers of peripheral inflammation and vascular dysfunction, only vascular dysfunction (not inflammation) was associated with cognitive impairment. Interestingly though, in an earlier meta-analysis authors [105] found no relationship between immune markers and cardiovascular surrogate outcomes in HIV infected individuals. A heterogeneity in methodology and a lack of follow-up were thought to be contributory factors. It is not unlikely there is a complex relationship between VRFs and the temporal and spatial impacts of HIV infection on the vascular system. We propose to assess peripheral markers of endothelial dysfunction, vascular permeability and pre-clinical CVD such as carotid intima thickness alongside inflammatory markers in future studies.

### Limitations

Our sample was relatively small. This is particularly relevant for vascular EOD markers such as stroke and MI with low overall reported case numbers. Our statistical examination of multiple vascular and demographic comparisons requires caution. Since we included individuals actively attending follow-up, there could be substantial survivorship bias, which is important given the high mortality reported with AF and MI in Tanzania [68,71]. Similarly, those with more severe cognitive impairment may have been more likely to default on follow-up.

Some VRFs were self-reported, limiting reliability. We also had limited information on treatment and adherence to treatment for hypertension and (self-reported) diabetes. Due to resource limitations, we were unable to complete carotid Doppler imaging. Similarly, we were unable to verify diabetes using HBAIC and given the potential role of inflammation in vascular risk, a peripheral inflammation measure, e.g., C reactive protein, would have been useful. We were unable to measure orthostatic hypotension as a marker of cerebral hypoperfusion. The MRI brain was only available for a subset (*n* = 90), and for the remainder (*n* = 63) prior stroke relied upon self-reporting and/or clinical examination findings. Some retinal images were of insufficient quality for hypertensive retinopathy grading other than arterial narrowing. When measuring vessel width to calculate AVR, the accurate identification of vessel edges was often challenging. A substantial subset of eyes and vessel pairs were excluded from the analysis due to rotation or poor quality. Furthermore, as vessels were measured at a set-point for repeatability, where a vessel pair divided early, AVR was potentially over- or under-estimated.

## 5. Conclusions

Contrary to our hypothesis, HAND appeared unrelated to traditional VRFs and EOD in this setting. Hypertension and hypertensive EOD were less prevalent than expected, compared with Tanzanian community data. Metabolic risk factors, including obesity and hypercholesterolaemia, were more prevalent than expected, but appeared unrelated to HAND. Given the study limitations, chronic inflammation and its potential contribution to endovascular dysfunction and vascular injury should be further explored.

## Figures and Tables

**Table 1:** Sociodemographic and HIV disease status characteristics of the baseline sample *n* = 153.

Sociodemographic Characteristics	
Females [*n* (%)]	103 (67.3)
Median age [years (IQR)]	56 (53–61.5)
Stratified Age Ranges [*n* (%)]	
50–54 years	55 (35.9)
55–59 years	42 (27.5)
60–64 years	32 (20.9)
65–70	15 (9.8)
70+ years	9 (5.9)
Highest education level [*n* (%)] (m = 1)	
0 years	8 (5.3)
1–4 years	18 (11.8)
5–7 years	104 (68.4)
More than 7 years	18 (11.8)
Higher education	4 (2.6)
Occupation [*n* (%)] (m = 4)	
Sedentary	44 (29.5)
Farmer	89 (59.7)
Other manual job	5 (3.4)
Not classified	3 (2.0)
Never worked	8 (5.4)
Currently in employment [*n* (%)] (m = 4)	140 (94.0)
Marital status [*n* (%)] (m = 3)	
Married	42 (28.0)
Widow(er)	61 (40.7)
Divorced	2 (1.3)
Separated	37 (24.7)
Never married	8 (5.3)
Household status [*n* (%)] (m = 3)	
Living alone	34 (22.7)
Living with spouse	27 (18.0)
Living with spouse and others	24 (16.0)
Living with family	60 (40.0)
Other	5 (3.3)
HIV disease characteristics	
Median time since HIV diagnosis [years (IQR)] (m = 1)	11.0 (6.0–13.0)
Mean current CD4 count ± SD [cells/mm3] (m = 23)	499.8 ± 269.6
Viral load (copies/mL) [*n* (%)] (m = 4)	
<20 20–10,000 >10,000	105 (70.5) 24 (16.1) 20 (13.4)
On cART treatment [*n* (%)]	152 (99.3)
cART regimen * [*n* (%)]	
First line Second line Not yet initiated	117 (76.5) 35 (22.9) 1 (0.7)
Forgets medication (self-report) [*n* (%)] (m = 1)	
Never Rarely Often Not applicable	136 (89.5) 13 (8.6) 2 (1.3) 1 (0.7)
Time off medication in the last 12 months (self-report) [*n* (%)] (m = 1)	
No Yes Not applicable	142 (93.4) 9 (5.9) 1 (0.7)
WHO clinical stage [*n* (%)] (m = 2)	
I II III IV	3 (2.0) 25 (16.6) 122 (80.8) 1 (0.7)
Previous CNS infection [*n* (%)] (m = 3)	4 (2.7)
TB infection status [*n* (%)] (m = 4)	
No infection Previous infection Current infection	125 (83.9) 23 (15.4) 1 (0.7)
Positive syphilis VDRL [*n* (%)] (m = 12)	23 (16.3)
Positive Hepatitis B [*n* (%)] (m = 11)	6 (4.2)
Positive Hepatitis C [*n* (%)] (m = 11)	1 (0.7)

* First-line regimens (NRTI x2 + NNRTI efavirenz/nevirapine) 1g-A (TDF, 3TC, EFV) 1b-A (AZT, 3TC, NVP/ABC,3TC, LPV/r), 1c-A(AZT, 3TC, EFV), 1e-A (TDF, FTC, EFV) 1f-A (TDF, FTC, NVP), 1h-A (TDF, 3TC, NVP), 1k-A (ABC, 3TC, EFV), 1m-A (ABC, 3TC, NVP), 1a-A (d4T, 3TC, NVP/d4T, 3TC, EFV), 1x-A (other 1st line unspecified). Second-line regimens (NRTI x2 + Protease Inhibitor (PI) 2f-A (TDF, FTC, LPV/r), 2h-A (TDF, FTC, ATV/r), 2s-A (AZT, 3TC, ATC/r), 2g-A (ABC, 3TC, LPV/r), 2e-A (TDF, 3TC, LPV/r), 2k-A (ABC/3TC, ATV/r), 2m-A (TDF, 3TC, ATV/r), 2n-A (AZT, 3TC, LPV/r/AZT, 3TC, EFV), 2x-A (other 2nd line unspecified).

**Table 2:** Prevalence of vascular risk factors and end-organ damage.

Vascular Risk Factors (VRFs)	
Median BMI [kg/m^2^ (IQR)]	22.9 (20.0–26.6)
BMI categories (kg/m^2^) [*n* (%)]	
Underweight (<18.5)	14 (9.2)
Normal (18.5–24.9)	82 (53.6)
Overweight (25.0–29.9)	41 (26.8)
Obese (≥30)	16 (10.5)
Mean WHR ± SD (m = 5)	0.873 ± 0.08
Abdominal obesity [*n* (%)] (m = 5)	81 (54.7)
Mean systolic BP ± SD (mmHg) (m = 1)	133.9 ± 27.3
Mean diastolic BP ± SD (mmHg) (m = 1)	80.72 ± 12.3
Hypertension [*n* (%)]	52 (34.0)
Diabetes Mellitus (self-report) [*n* (%)] (m = 3)	8 (5.3)
If DM, on treatment [*n* (%)]	
Yes	6 (75.0)
No	1 (12.5)
Defaulted from treatment	1 (12.5)
Smoking status [*n* (%)]	
Never smoked	113 (73.9)
Previously smoker	33 (21.6)
Current smoker	7 (4.6)
Alcohol consumption [*n* (%)] (48 values missing)	
Never	17 (16.2)
Previous	31 (29.5)
Occasional	31 (29.5)
Regular	26 (24.8)
Mean serum cholesterol ± SD [mmol/L] (m = 12)	4.7 ± 1.2
Hypercholesterolaemia [*n* (%)] (m = 12)	47 (33.3)
End Organ Damage (EOD)	
Atrial fibrillation [*n* (%)] (m = 1)	0 (0.0)
Previous stroke [*n* (%)]	8 (5.2)
Prior MI [*n* (%)] (m = 3)	2 (1.3)
LVH [*n* (%)] (m = 1)	19 (12.5)
Proteinuria [*n* (%)] (m = 1)	5 (3.3)
Median serum creatinine [μol/L (IQR)]	66.0 (56.0–77.5)
CKD [*n* (%)] (m = 12)	8 (5.7)
Mean ABPI ± SD	1.1 ± 0.2
PAD [*n* (%)] (m = 3)	5 (3.3)
Arterial stiffening [*n* (%)] (m = 3)	6 (4.0)
Median pulse pressure [mmHg (IQR)]	49.0 (39.0–62.0)

**Table 3:** Vascular risk factors by age band.

Risk Factor	50–59 Years (*n* = 97)		≥60 Years (*n* = 56)		Chi-Squared Test
	Prevalence (%)	95% CI	Prevalence (%)	95% CI	
Obesity	11.3	5.0–17.7	8.9	1.5–16.4	X^2^= 0.221, *p* = 0.639
Abdominal obesity	54.7 (m = 2)	44.7–64.7	54.7 (m = 3)	41.3–68.1	X^2^ = <0.0001, *p* = 0.998
Hypertension	26.8	18.0–35.6	46.4	33.4–59.5	X^2^ = 6.094, *p* = 0.014 **
Diabetes mellitus	4.2 (m = 2)	0.2–8.2	7.3 (m = 1)	0.4–14.1	*p* = 0.465 (Fisher’s exact test)
Smoker (current/previous)	22.7	14.3–31.0	32.2	19.9–44.4	X^2^ = 1.646, *p* = 0.199
Current alcohol	50.0 (m = 27)	38.3–61.7	62.9 (m = 21)	46.8–78.9	X^2^ = 1.554, *p* = 0.213
Hypercholesterolaemia	38.5 (m = 6)	28.5–48.5	24.0 (m = 6)	12.2–35.8	X^2^ = 3.037, *p* = 0.081

** Significant associations.

**Table 4:** Vascular risk factors and markers of end organ damage in individuals with and without HAND.

Variable	HAND (*n* = 102)	No HAND (*n* = 51)	Statistical Comparison
Vascular risk factors (VRFs)			
Median BMI [kg/m^2^ (IQR)]	21.9 (19.7–25.8)	23.7 (20.0–27.1)	U = 2164.5, Z = −1.689, *p* = 0.091
Obesity [*n* (%)]	10 (9.8)	6 (11.8)	X^2^= 0.140, *p* = 0.709
Mean WHR ± SD	0.87 ± 0.09 (m = 3)	0.88 ± 0.06 (m = 2)	T = 1.173, *p* = 0.243
Abdominal obesity [*n* (%)]	50 (50.5) (m = 3)	31 (63.3) (m = 2)	X^2^ = 2.154, *p* = 0.142
Mean systolic BP ± SD (mmHg)	132.4 ± 27.5	137.0 ± 26.7 (m = 1)	T = 0.972, *p* = 0.333
Mean diastolic BP ± SD (mmHg)	79.2 ± 12.0	83.9 ± 12.5 (m = 1)	T = 2.247, *p* = 0.026 **
Hypertension [*n* (%)]	32 (31.4)	20 (39.2)	X^2^ = 0.932, *p* = 0.334
Diabetes mellitus [*n* (%)]	6 (6.0) (m = 2)	2 (4.0) (m = 1)	*p* = 0.719 (Fisher’s exact test)
Smoker (current/previous) [*n* (%)]	28 (27.5)	12 (23.5)	X^2^ = 0.271, *p* = 0.603
Current alcohol consumption [*n* (%)]	35 (55.6) (m = 39)	22 (52.4) (m = 9)	X^2^ = 0.102, *p* = 0.749
Mean serum cholesterol ± SD [mmol/L]	4.7 ± 1.3 (m = 6)	4.9 ± 1.1 (m = 6)	T = 0.908, *p* = 0.365
Hypercholesterolaemia [*n* (%)]	31 (32.3) (m = 6)	16 (35.6) (m = 6)	X^2^ = 0.147, *p* = 0.702
Vascular End organ damage (EOD)			
Previous stroke [*n* (%)]	5 (4.9)	3 (5.9)	*p* = 1.000 (Fisher’s exact test)
Prior MI [*n* (%)]	1 (1.0)	1 (2.1) (m = 3)	X^2^ = 0.302, *p* = 0.583
LVH [*n* (%)]	10 (9.9) (m = 1)	9 (17.6)	X^2^ = 1.859, *p* = 0.173
Proteinuria [*n* (%)]	4 (4.0) (m = 1)	1 (2.0)	*p* = 0.454 (Fisher’s exact test)
CKD [*n* (%)]	7 (7.3) (m = 6)	1 (2.2) (m = 6)	*p* = 0.211 (Fisher’s exact test)
Median creatinine [μol/L (IQR)]	66.0 (56.0–77.75) (m = 6)	68.0 (56.0–76.5) (m = 6)	U = 2220.5, Z = 0.268, *p* = 0.789
Mean ABPI ± SD	1.08 ± 0.19 (m = 3)	1.08 ± 0.12	T = −0.250, *p* = 0.803
PAD [*n* (%)]	4 (4.0) (m = 3)	1 (2.0)	*p* = 0.445 (Fisher’s exact test)
Arterial stiffening [*n* (%)]	4 (4.0) (m = 3)	2 (3.9)	*p* = 0.669 (Fisher’s exact test)
Median pulse pressure [mmHg (IQR)]	48.0 (38.0–61.3)	49.0 (40.0–64.0)	X2 = 2557.5, Z = −0.168, *p* = 0.866
Mean AVR ± SD	0.76 ± 0.08 (m = 25)	0.73 ± 0.08 (m = 7)	T = −1.587, *p* = 0.115
WMD rating [*n* (%)] None Mild Moderate Severe	(m = 45)	(m = 18)	U = 937, Z = −0.031, *p* = 0.975
	26 (45.6)	13 (39.4)	
	9 (15.8)	9 (27.3)	
	21 (36.8)	11 (33.3)	
	1 (1.8)	0 (0.0)	

** Significant associations.

## Data Availability

Anonymised data associated with this study will be made available upon reasonable request to the study authors, subject to approval procedures of the Tanzanian institute of medical research (NIMR) and where necessary the completion of a Data Transfer Agreement.

## Supplementary Materials

The following supporting information can be downloaded at: https://www.mdpi.com/article/10.3390/v16060819/s1, Figure S1: Neuropsychological test battery utilised in this study and associated validation data; Table S1: Sociodemographic and HIV disease status characteristics in those with and without HAND; Table S2: Sociodemographic, HIV disease severity, vascular risk factors and cardiovascular end organ damage markers in individuals with and without symptomatic HAND Tables S3 and S4: Multivariable models for primary and secondary outcome.
