# Supplementary material for: Lack of Association of Vascular Risk Factors with HIV-Associated Neurocognitive Disorders in cART-Treated Adults Aged ≥ 50 Years in Tanzania
Source: Viruses. 2024 May 22;16(6):819. doi: 10.3390/v16060819 (PMC11209468; doi:10.3390/v16060819)
Supplement: Supplementary file 1 [file viruses-16-00819-s001.zip › viruses-2839843-supplementary.pdf]

Supplementary Online Data

Supplementary Table S1: Sociodemographic and HIV disease status characteristics in those with and without HAND.

Variable	HAND (n=102)	No HAND (n=51)	Statistical comparison[1]
**Median Age [years (IQR)]**	55.5 (53.0-62.0)	58.0 (54.0-61.0)	U=2378.0, Z=-0.865, p=0.387
**Females [n (%)]**	69 (67.6)	34 (66.7)	X^2=0.015, p=0.903
**Highest educational attainment [n (%)]**			X^2=3.332, p=0.189
≤4 years	18 (17.6)	9 (17.6)	
5-7 years	73 (71.6)	31 (60.8)	
>7 years	11 (10.8)	11 (21.6)	
**Median time since HIV diagnosis [years (IQR)]**	11.0 (6.0-13.0) (m=1)	11.0 (6.0-13.0) (m=2)	U=2423.0, Z=-0.208, p=0.836
**Median nadir CD4 count [cells/mm^3 (IQR)]**	177.5 (103.8-271.3)	209.0 (109.5-294.5) (m=1)	U=2367.0, Z=-0.718, p=0.473
**Mean current CD4 count ± SD [cells/mm^3]**	493.2 ± 268.4 (m=15)	513.3 ± 274.6 (m=8)	T=0.399, p=0.691
**Undetectable viral load**	71 (71.7) (m=3)	34 (68.0) (m=1)	X^2=0.221, p=0.639
**WHO clinical stage [n (%)]**	(m=1)	(m=1)	X^2=5.237, p=0.155
I	2 (2.0)	1 (2.0)	
II	13 (12.9)	12 (24.0)	
III	86 (85.1)	36 (72.0)	
IV	0 (0.0)	1 (2.0)	
**2nd line cART regimen [n (%)]**	20 (19.8) (m=1)	15 (29.4)	X^2=1.766, p=0.184
**History CNS infection**	2 (2.0) (m=3)	2 (3.9)	P=0.605 (Fisher's exact test)
**History TB infection**	17 (17.0) (m=2)	7 (14.3) (m=2)	X^2=0.179, p=0.814
**Positive hepatitis B serology [n (%)]**	4 (4.2) (m=7)	2 (4.3) (m=4)	P=1.000 (Fisher's exact test)
**Positive hepatitis C serology [n (%)]**	1 (1.1) (m=7)	0 (0.0) (m=4)	P=1.000 (Fisher's exact test)
**Positive syphilis VDLR [n (%)]**	15 (16.0) (m=8)	8 (17.0) (m=4)	X^2=0.026, p=0.872

[1] Statistical tests used: Mann–Whitney U, Pearson-chi squared, Fisher's exact test, T-test

Supplementary Data Table S2: Sociodemographic, HIV disease severity, vascular risk factors and cardiovascular end organ damage markers in individuals with and without symptomatic HAND.

Variable	Symptomatic HAND (n=39)	No symptomatic HAND (n=114)	Statistical comparison
**Median Age [years (IQR)]**	57.0 (52.0-68.0)	56.0 (53.0-61.0)	U=2414.0, Z=0.801, p=0.423
**Females [n (%)]**	29 (74.4)	74 (64.9)	X^2=1.179, p=0.278
**Highest education grade [n (%)]**<br>**≤4 years**<br>**5-7 years**<br>**More than 7 years**	 10 (25.6)<br>23 (59.0)<br>6 (15.4)	 17 (14.9)<br>81 (71.1)<br>16 (14.0)	 X^2=2.556, p=0.279
**Median time since HIV diagnosis [years (IQR)]**	9.5 (6.0-12.0) (m=1)	11.0 (6.0-13.0) (m=2)	U=1985.5, Z=-0.619, p=0.536
**Median nadir CD4 count [cells/mm^3 (IQR)]**	207.0 (112.0-395.0)	170.0 (104.5-268.0) (m=1)	U=2509.0, Z=1.289, p=0.197
**Mean current CD4 count ± SD [cells/mm^3]**	584.0 ± 264.7 (m=9)	474.6 ± 267.2 (m=14)	t=-1.971, p=0.051
**Undetectable viral load**	25 (65.8) (m=1)	80 (72.1) (m=3)	X^2=0.537, p=0.464
**WHO clinical stage [n (%)]**		(m=2)	U=2112.5, Z=-0.444, p=0.657
**I**	1 (2.6)	2 (1.8)	
**II**	5 (12.8)	20 (17.9)	
**III**	33 (84.6)	89 (79.5)	
**IV**	0 (0.0)	1 (0.9)	
**2nd line cART regimen [n (%)]**	10 (25.6)	25 (22.1) (m=1)	X^2=0.202, p=0.653
**History CNS infection**	1 (2.6) (m=1)	3 (2.7) (m=2)	P=1.000 (Fisher's exact test)
**History TB infection**	8 (20.5)	16 (14.5) (m=3)	X^2=0.759, p=0.384
**Positive hepatitis B serology [n (%)]**	1 (2.7) (m=2)	5 (4.8) (m=9)	P=1.000 (Fisher's exact test)
**Positive hepatitis C serology [n (%)]**	2 (5.1)	1 (1.0) (m=9)	P=1.000 (Fisher's exact test)
**Positive syphilis VDLR [n (%)]**	9 (25.0) (m=3)	14 (13.3) (m=9)	X^2=2.673, p=0.102
**Vascular risk factors (VRFs)**			
**Median BMI [kg/m^2 (IQR)]**	21.5 (19.3-25.5)	22.9 (20.2-27.1)	U=1834.0, Z=-1/629, p=0.103
**Obesity [n (%)]**	2 (5.1)	14 (12.3)	p=0.362 (Fisher's exact test)
**Mean WHR ± SD**	0.86 ± 0.07 (m=1)	0.88 ± 0.09 (m=4)	T=0.772, p=0.442
**Abdominal obesity [n (%)]**	22 (57.9) (m=1)	59 (53.6) (m=4)	X^2=2.07, p=0.649
**Mean systolic BP ± SD (mmHg)**	131.7 ± 26.9	134.7 ± 27.5 (m=1)	T=0.595, p=0.553
**Mean diastolic BP ± SD (mmHg)**	77.3 ± 11.9	81.9 ± 12.3 (m=1)	T=0.950, p=0.046**
**Hypertension [n (%)]**	13 (33.3)	39 (34.2)	X^2=0.1, p=0.921
**Diabetes mellitus [n (%)]**	4 (10.5) (m=1)	4 (3.6) (m=2)	X^2=2.718, p=0.099
**Smoker (current/previous) [n (%)]**	10 (25.6)	30 (26.3)	X^2=0007, p=0.934
**Currently drinks alcohol [n (%)]**	12 (66.7) (m=21)	45 (51.7) (m=27)	X^2=1.342, p=0.247
**Mean serum cholesterol ± SD [mmol/L]**	4.3 ± 0.9 (m=1)	4.9 ± 1.3 (m=11)	T=2.342, p=0.021**
**Hypercholesterolaemia [n (%)]**	10 (26.3) (m=1)	37 (32.5) (m=11)	X^2=1.153, p=0.283
**Vascular end organ damage (EOD)**			
**Previous stroke [n (%)]**	2 (5.1)	6 (5.3)	P=1.000 (Fisher's exact test)
**Prior MI [n (%)]**	1 (2.6)	1 (0.09) (m=3)	P=0.454 (Fisher's exact test)
**LVH [n (%)]**	3 (7.7)	16 (14.2) (m=1)	P=0.404 (Fisher's exact test)
**Proteinuria [n (%)]**	2 (5.3) (m=1)	3 (2.6)	P=0.599 (Fisher's exact test)
**CKD [n (%)]**	4 (10.5) (m=1)	4 (3.9) (m=11)	P=0.211 (Fisher's exact test)
**Median creatinine [umol/L (IQR)]**	66.0 (56.5-80.0) (m=1)	66.0 (56.0-76.0) (m=11)	U=1996.5, Z=0.184, p=0.854

**Mean ABPI ± SD**	1.08 ± 0.10	1.08 ± 0.18<br>(m=3)	T=0.102, p=0.919
**PAD [n (%)]**	1 (2.6)	4 (3.6)<br>(m=3)	P=1.000<br>(Fisher's exact test)
**Arterial stiffening [n (%)]**	1 (2.6)	5 (4.5)<br>(m=3)	P=1.000<br>(Fisher's exact test)
**Median pulse pressure [mmHg (IQR)]**	46.0 (41.0-66.0)	49.0 (38.8-62.0)	U=2315.0, Z=0.385, p=0.700
**Mean AVR ± SD**	0.75 ± 0.09<br>(m=14)	0.75 ± 0.08<br>(m=18)	T=-0.342, p=0.747
**WMD rating [n (%)]**	(m=21)	(m=42)	U=548.5, Z=-1.079, p=0.281
**None**	6 (33.3)	33 (45.8)	
**Mild**	4 (22.2)	14 (19.4)	
**Moderate**	7 (38.9)	25 (34.7)	
**Severe**	1 (5.6)	0 (0.0)	

** significant findings

Supplementary Data S3 and S4. Multivariable models for primary and secondary outcome.

Multivariable model HAND vs. no HAND (primary outcome).

	B	SE	Wald	df	Sig	Exp (B)	Lower	Upper
**sex**	-0.83	0.374	0.050	1	0.824	0.920	0.442	1.915
**age**	-0.02	0.028	0.006	1	0.939	0.998	0.945	1.053
**Diastolic BP**	-0.031	.014	4.722	1	0.030	0.969	0.943	0.997

A. Variables entered on step 1, sex, age, diastolic BP

Multivariable model symptomatic HAND vs. no symptomatic HAND.

	B	S.E	Wald	df	Sig	Exp (B)	Lower	Upper
**sex**	-0.633	0.447	2.003	1	0.157	0.531	0.221	1.276
**age**	.059	.031	3.779	1	0.052	1.061	1.00	1.127
**Diastolic BP**	-0.28	.018	2.348	1	0.125	0.972	0.938	1.008
**Total serum cholesterol**	-.405	.207	3.819	1	0.051	0.667	.444	1.001

Supplementary Data Figure S1: Neuropsychological test battery utilised in this study and associated validation data.

Test item and measure recorded	Relevant validation and reference studies	Cognitive domain And subdomains	Brief description (measures utilised in this study)
**WHO-UCLA Auditory Verbal Learning Test (AVLT)+***<br><br>**Total correct/trial**	Validation (HIV)<br><br>SSA (Kenya, Zaire)1,3<br><br>Reference study (2)	**Verbal memory**<br>Verbal learning<br>Immediate recall<br>Post interference recall<br>30 minute delayed recall<br>Recognition memory	15 word learning list.<br>Trials 1-5 (learning)<br>Trial 5 (immediate recall)<br>Trial 7 (recall post interference (unrelated) word list)<br>Trial 8 (30 minute delayed recall)
**Grooved pegboard +**<br>**(dominant/non dominant hand)**<br><br>**Time (seconds)**<br>**Dominant**<br>**Non-dominant hand**	Validation (HIV) (4,5)<br>Uganda<br>Brazil (low-literacy)<br><br>Reference study (1-3)	**Psychomotor speed**<br><br>Fine motor<br>2D spatial	Insertion of 25 metal pegs into identical but differently orientated holes in order.<br><br>Errors highlighted by examiner.
**10m Timed walk +****<br><br>**Mean time (seconds) over 3 trials**	Reference study<br><br>SSA (Kenya, Zaire<br>(1-3)	***Motor speed	Timed walk along a measured 10 metre distance. Examiner records time in seconds from markers at 2 metres and 8 metres over 3 attempts.
**Orientation from Tanzanian Alzheimer's Disease Assessment Scale-cognitive subscale (ADAS-cog)++**<br>**Total correct**	Validation (non-HIV, low literacy)<br><br>Tanzania (6)	Orientation	Name, day, date, month, year, time of day (nearest hour) and place.<br><br>Scored out of 7.
**Colour trails 1+**<br>**(low literacy variant of the trail making test)**<br><br>**Time to complete (seconds)**	Validation (HIV)<br><br>SSA (Kenya, Zaire)<br><br>Reference study (1-3)	**Psychomotor speed**<br>Sustained attention	Marking a trail of numbers 1-25 in sequence in pencil without lifting the pencil from the page
**Colour trails 2+**<br>**(low literacy variant of the trail making test)**<br><br>**Time to complete (seconds)**	Validation (HIV)<br><br>SSA (Kenya, Zaire)<br><br>Reference study (1-3)	**Executive function**<br>Processing speed<br>Selective attention	Sequencing of numbers 1-25 as in Color Trails 1 whilst alternating pink and yellow colours.<br><br>Errors highlighted by examiner.
**Digit span +**<br><br>**Longest correct span 2-9**	Validation (HIV)<br><br>SSA (Kenya, Zaire)	**Working memory**<br>Forwards<br>Backwards<br>Total	Progressively increasing number lists from 2 digits onwards repeated by participant until failure.

	Reference study (1-3)		
**Stick design test ++**<br><br>**Correct elements as per score guide**<br><br>**NB forms part of Tanzanian Alzheimer's Disease Assessment Scale-cognitive subscale (ADAS-cog)**	Validation (7)<br>Nigeria, Brazil (low-literacy)[101] 7,8)<br>Non-HIV cognitive impairment (Tanzania) (6)	Visuoconstruction<br><br>Attempt 1<br>Visual memory<br><br>Attempt 2<br>Visuoconstruction	Reconstruction of four previously demonstrated matchstick shapes. Scoring guidance as in<br><br>Maximum score 12
**Categorical verbal fluency*****<br><br>**Number of correct items/minute**	Adapted from 'supermarket task'<br>Validations (low-literacy)<br>Brazil<br>Turkish immigrants<br>(9-12)	Verbal fluency****	Items typically found in market listed in one minute.<br>Inappropriate items and repeats recorded for additional clinical information (evidence of perseveration etc)
**Verbal commands from Tanzanian Alzheimer's Disease Assessment Scale-cognitive subscale (ADAS-cog) ++**<br>**Total trials correct**	Validation (non-HIV, low literacy)<br><br>Tanzania (6)	Verbal language comprehension	Completion of five verbal commands of increasing complexity

Additional confirmatory bedside tests

(NB not normed, but available as supportive information to aid clinical judgement).

Three item registration and recall, subjective rating of expressive language, reciprocal alternating movements, Luria three hand position test, praxis (strike match, demonstrate toothbrush use)

+ Included within original WHO neuropsychological test battery for HIV

++ Additional measures of cortical function locally validated

*Culture-fair adaptation of the Rey Auditory Verbal Learning Test (AVLT)

** Not utilised for HAND criteria, but provided additional clinical information to aid clinical judgement

***Adaptation of the 'supermarket item' naming task (reportedly less educationally/culturally influenced than animal naming task). NB for references related to challenges of phonemic (letter) fluency in low-literacy settings supportive use of categorical fluency in this setting see.

Footnote: Key References:

<u>Colour trails 1 and 2, WHO-UCLA AVLT</u>

1 Maj M, Janssen R, Satz P, **et al.** ***The World Health Organization's Cross-cultural Study on Neuropsychiatric Aspects of Infection with the Human Immunodeficiency Virus 1 (HIV-1) Preparation and Pilot Phase. The British Journal of Psychiatry. 1991 Sep;159(3):351-6.***

2 Maj M, Satz P, Janssen R, Zaudig M, **et al.** ***G. WHO Neuropsychiatric AIDS Study, cross-sectional phase II: neuropsychological and neurological findings. Archives of general psychiatry. 1994 Jan 1;51(1):51-61.***

3 Maj M, D'Elia L, Satz P, Janssen R**et al.** ***Evaluation of two new neuropsychological tests designed to minimize cultural bias in the assessment of HIV-1 seropositive persons: a WHO study. Archives of clinical Neuropsychology. 1993 Jan 1;8(2):123-35.***

<u>Pegboard brazil low literacy (validation)</u>

4 de Almeida SM, Kamat R, Cherner M, et al. *Improving detection of HIV-associated cognitive impairment: comparison of the international HIV dementia scale and a brief screening battery. Journal of acquired immune deficiency syndromes (1999). 2017 Mar 1;74(3):332.*

Uganda – validation for HIV dementia

5 Sacktor NC, Wong M, Nakasujja N, et al. *The International HIV Dementia Scale: a new rapid screening test for HIV dementia. Aids. 2005 Sep 2;19(13):1367-74.*

<u>Orientation and verbal commands</u>

6. Paddick, S.M., et al.*, Adaptation and validation of the Alzheimer's Disease Assessment Scale - Cognitive (ADAS-Cog) in a low-literacy setting in sub-Saharan Africa. Acta Neuropsychiatr, 2017: p. 1-8.*

Visuoconstruction (stick design test)

7. Baiyewu, O., et al.*, The Stick Design test: A new measure of visuoconstructional ability. Journal of the International Neuropsychological Society, 2005. 11(5): p. 598-605.*

8. de Paula JJ, Costa MV, Bocardi MB, et al. *The Stick Design Test on the assessment of older adults with low formal education: evidences of construct, criterion-related and ecological validity. International psychogeriatrics. 2013 Dec;25(12):2057-65.*

<u>Market test</u>

9.Nielsen TR, Waldemar G. Effects of literacy on semantic verbal fluency in an immigrant population. Aging Neuropsychol Cogn. 2016;23(5):578-90.

10.Neves TR, Araújo NB, Silva FD, et al. *Accuracy of the semantic fluency test to separate healthy old people from patients with Alzheimer's disease in a low education population. Journal Brasileiro de Psiquiatria. 2020 Jun 1;69:82-7.*

11. Nielsen TR, Waldemar G. Effects of literacy on semantic verbal fluency in an immigrant population. Aging, Neuropsychology, and Cognition. 2016 Sep 2;23(5):578-90.

<u>Verbal fluency</u>

12. Ratcliff G, Ganguli M, Chandra V, et al. *Effects of literacy and education on measures of word fluency. Brain and Language. 1998 Jan 1;61(1):115-22.*
